# The association between fear extinction, the ability to accomplish exposure and exposure therapy outcome in specific phobia

**DOI:** 10.1038/s41598-020-61004-3

**Published:** 2020-03-09

**Authors:** Friederike Raeder, Christian J. Merz, Jürgen Margraf, Armin Zlomuzica

**Affiliations:** 1 Mental Health Research and Treatment Center, Ruhr University Bochum, Bochum, Germany; 2 Department of Cognitive Psychology, Ruhr University Bochum, Bochum, Germany

**Keywords:** Human behaviour, Anxiety

## Abstract

Great interest exists in maximizing exposure therapy efficacy in anxiety disorders. At the same time, reduced frequency and shortened duration of exposure sessions are required to meet the specific regularities in routine care settings. Extinction has emerged as the key mechanism of exposure treatment in anxiety disorders. Examining exposure treatment processes from the perspective of extinction learning might provide novel insights into variability in exposure treatment duration and outcome. The present study sought to examine the functional link between fear extinction, the ability to accomplish exposure in a predetermined time and exposure therapy outcome in specific phobia. Treatment-seeking individuals (*N* = 53) with spider phobia underwent a context-dependent fear conditioning paradigm prior to a standardized exposure. Spider-phobic participants who were able to complete exposure within the pre-determined time (i.e., completers) showed a more pronounced short- and long-term exposure therapy benefit. In the fear conditioning task, a more pronounced decline in CS-US contingency ratings during extinction (retrieval) was found in completers relative to non-completers. The failure to further extinguish US expectancy to the CSs in non-completers might offer a potential mechanistic explanation why non-completers have difficulties to accomplish all exposure steps in a fixed time and show less pronounced treatment gains. Our findings bear specific implications for the implementation of exposure treatment to routine care settings.

## Introduction

Exposure is considered as the treatment of choice for anxiety disorders. Exposure therapy is aimed at helping patients to overcome their anxiety by creating a safe environment, in which they are repeatedly and systematically exposed to feared or avoided scenarios, leading to decreases of fear^1,2^. Despite its effectiveness, some patients show no or only a partial remission of symptoms or experience a recovery of symptoms after treatment^3^. Likewise, some patients exhibit a delayed therapeutic benefit, requiring an extended treatment duration and frequency^4^. This, however, is at odds with the organizational and economic demands in routine care, which requires efficient time schedules and reduction of treatment-associated costs^5^. The frequency and duration of exposure sessions thus needs to be adapted to the specific regularities in routine care settings (e.g. see^6^). The identification of new pathways to improve exposure treatment efficacy while at the same time decreasing barriers to transfer exposure treatment to routine care would be highly desirable^7^. So far, only little is known about mechanisms contributing to treatment duration and short- and long-term treatment outcome.

Extinction has emerged as a central candidate for the main process underlying exposure^8^. Consequently, research on the cognitive and neurobiological foundations of extinction as the laboratory analog of exposure might pave the way to understand and improve exposure treatment efficacy. Ultimately, by bridging the gap between the laboratory and the clinic^9^, it might guide individualized treatment options for anxiety disorders, which are derived from a mechanism-based understanding of extinction^10^.

Despite the strong evidence for an implication of the extinction model in the etiology and treatment of anxiety disorders^11^, very few studies have examined the mutual relationship between extinction learning performance and exposure therapy outcome^12–14^. Waters and Pine^12^ assessed fear conditioning in anxious children who responded well and children who did not respond to cognitive behavioral therapy (CBT). CBT involved a set of different program modules such as psychoeducation, relaxation techniques, cognitive reconstructuring, social skills training, exposure and other techniques. They showed that anxious children, who were diagnosis‐free after CBT, displayed an efficient physiological decline to the CSs during extinction (similar to a group of non-anxious children who constituted a control group). In contrast, anxious children, who retained diagnoses after CBT, tended to acquire more negative evaluations of both CSs and failed to show reductions in physiological responses to the CSs during extinction. While in this pioneering work of Waters and Pine^12^ the relation between CBT-success in general (defined as the absence of a diagnosis after therapy completion) and fear conditioning was assessed in children, two other studies analyzed the predictive value of fear conditioning measures to standardized exposure benefit in adults. Forcadell *et al*.^13^ provided evidence for an association between fear extinction and the outcomes of an analog of exposure therapy. Individuals with fear of spiders showed higher extinction learning capacity (based on the discrimination ratio between the CS+ and CS−) and exhibited greater fear reduction from pre- to post- exposure therapy analog. Fear conditioning was assessed on different levels. Notably, positive associations with therapy outcome were not found across all fear measures. Ball *et al*.^14^ demonstrated that better extinction learning (less negative stimulus ratings) in addition to a specific pattern of neural activation (i.e., greater activation in ventromedial prefrontal cortex and less activation in amygdala) was linked to greater self-reported anxiety reduction two weeks after exposure in individuals with public speaking anxiety.

In conclusion, the methodology, direction and pattern of associations between fear conditioning measures and exposure outcome measures varies greatly and  warrants further replication. In terms of methodology, some studies related different fear conditioning measures to various treatment outcome measures to derive meaningful associations^13,14^. This approach might be difficult for several reasons: Fear conditioning can be assessed on different fear system levels, e.g., by using physiological, valence and/or contingency measures. Further, various differences in the operationalization of fear acquisition, extinction and/or extinction retrieval exist^15^. Thus, numerous associations between different conditioning measures (e.g., CS+ responses or CS+ versus CS− responding during fear acquisition or fear extinction or extinction retrieval, etc.) and treatment-related markers (e.g., changes in self-report fear, avoidance behavior, etc.) are possible. This can potentially lead to false positive correlations. It remains challenging to interpret which of these measures are “more valid” and how these measures can contribute to therapy processes on a more mechanistic level. Alternatively, one could characterize participant’s fear conditioning signatures by using dichotomous independent variables (see^12^). While this approach is also not without pitfalls, it may be less susceptible to the biasing influence of researcher degrees of freedom than the selection of prognostic measures from fear conditioning measures.

In addition, the existing studies so far did not investigate the role of contextual factors mediating the link between extinction and exposure therapy. Context plays an important role during both acquisition and retrieval of extinction memories^16^. The demonstration that conditioned fear responses can reoccur after extinction learning when an excitatory CS is presented in an unfamiliar context (fear renewal)^16^ is of high clinical interest as it might correspond to one form of relapse after exposure^16–18^.

Furthermore, none of the studies so far investigated whether changes in fear conditioning might be linked to the ability to complete exposure therapy in a predetermined time. The latter might be of special importance when implementing exposure in routine care settings.

In the present study, spider-phobic individuals seeking treatment were subjected to a context-dependent differential fear conditioning task in virtual reality^19^. Fear conditioning was followed by a standardized exposure without further cognitive-therapeutic interventions. We examined whether the ability to complete all exposure steps^20^ and treatment efficacy can be traced back to systematic changes in conditionability, particularly fear extinction learning and retrieval. We hypothesized that an enhanced extinction learning and retrieval might be linked to the ability to complete the exposure within a predetermined time. We further expected that the ability to accomplish exposure should affect short- and long-term exposure therapy outcome. As an extended measure of therapy outcome, we attempted to assess the mediating role of context after treatment completion.

## Materials and Method

### Participants

Spider-phobic participants were recruited via various bulletin boards postings in social media networks. Exclusion criteria comprised any neurological condition, the use of high-dose medication, alcohol or drug abuse and being able to reach a Behavioral Approach Test (BAT) score of 10 at pre-treatment, with the latter leading to an exclusion of *n* = 6 post-testing. Our final sample comprised *N* = 53 spider-phobic participants. All experimental procedures were approved by the local ethics committee of the Ruhr University Bochum and were carried out in accordance with the principles outlined by the Declaration of Helsinki. All participants provided written informed consent and could receive 6 course credits for their participation if applicable.

### Experimental design and procedure

**Figure 1:**
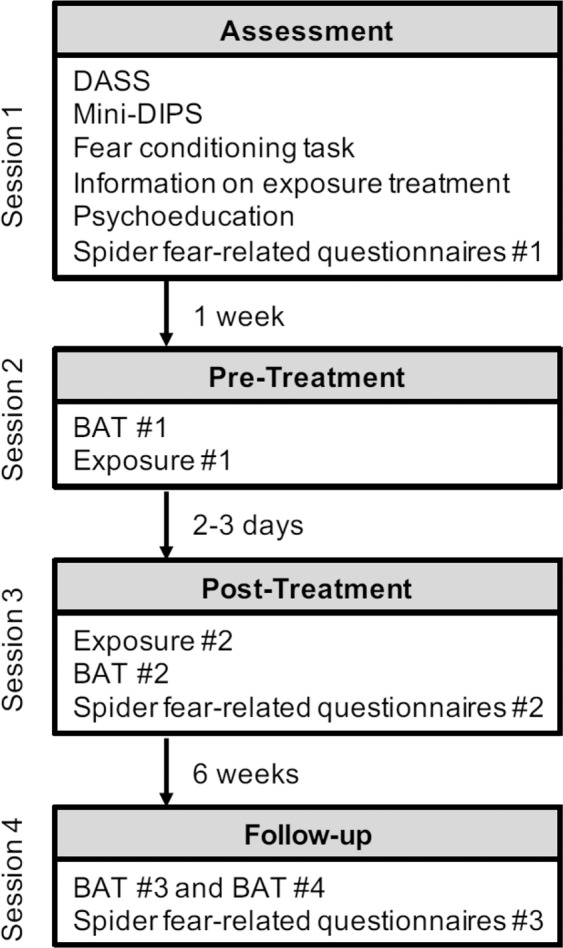
Graphic illustration of the experimental design and procedure. Participants were subjected to four sessions. At session 1, participants completed the DASS^23^, the Mini-DIPS^24^ and the fear conditioning task^19^. At the end of the session, participants received information about the exposure treatment, psychoeducation about spider phobia, and filled in the spider-fear related questionnaires (#1). At session 2 (conducted approx. 1 week later), participants received instructions on the BAT and were familiarized with the SUDS. Participants then completed the first BAT (#1), after which the first exposure session was conducted. Approx. 2-3 days later, at session 3, the second exposure session was conducted, followed by a short rehearsal of BAT instructions and the completion of the BAT (#2) and the spider-fear related questionnaires (#2). The follow-up assessment (session 4) was conducted approximately six weeks later. Here, the BAT (#3) was conducted in both the treatment context as well as in a novel context (BAT #4), with a randomized order of context presentation. At the end of session 4, the spider-fear related questionnaires (#3) were completed and participants were fully debriefed. DASS Items: Depression Anxiety Stress Scales^23^.

The experimental design and its associated procedure are depicted in chronological order in Fig. 1. Briefly, at session 1, the fear conditioning task was conducted. Exposures were conducted at session 2 and session 3. As a measure of therapy outcome, BATs and spider-fear related questionnaires were completed prior to the first exposure, after the second exposure session and at session 4 (i.e., six weeks follow-up). Of note, at session 4, the BAT was conducted two times, i.e., in the former therapeutic context and a novel context, to assess contextual renewal.

### Fear conditioning

The differential conditioning paradigm is described in detail in Mosig *et al*.^19^. Briefly, the reinforced (CS+) and unreinforced (CS−) conditioned stimuli were a high (300 Hz) and a low frequency (135 Hz) tone, which were presented for 8 s via headphones (60 dB). CSs were counterbalanced across participants and presented in pseudorandom order. The unconditioned stimulus (US) comprised a 500 ms mild electric stimulation delivered to the skin of the lower arm via Ag/AgCl electrodes. The intensity of the US was individualized for each participant, i.e., adjusted to a sensation level that was perceived as highly unpleasant but not painful. Presentation software (Neurobehavioural Systems, USA) controlled stimulus delivery. The inter-trial-interval (ISI) varied randomly between 18 and 22 s.

**Figure 2:**
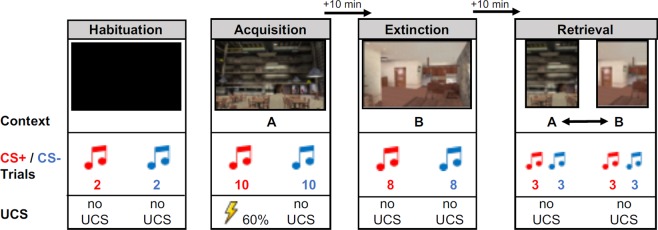
The experimental design of the context-dependent differential fear conditioning procedure. The fear conditioning task utilized an AB(AB) renewal setup in virtual reality, which involved the presentation of two contexts (A and B; created with Max Payne software) via a 3D head-mounted display (Z800, eMagin, USA). Contexts featured a cafeteria (A) and an apartment (B). Context presentation during the fear acquisition and extinction training phases as well as the order of context presentation during the retrieval test (indicated by double arrows) was counterbalanced across participants. Breaks of 10 minutes were imposed after fear acquisition and extinction training.

The fear conditioning task utilized an AB(AB) renewal setup. The contexts depicted a cafeteria or a room, with their assignment to context A or B being counterbalanced across participants. As displayed in Fig. 2, it comprised habituation (2 CS+, 2 CS−), during which a black screen was presented, fear acquisition training in context A (10 CS+, 10 CS−), extinction training in context B (8 CS+, 8 CS−), and a retrieval test in both the former acquisition context A (3 CS+, 3 CS−) and the extinction context B (3 CS+, 3 CS−). The order of context presentation during the retrieval test was randomized between participants. The CS+ co-terminated with the US on a 60% reinforcement schedule only during fear acquisition training. The CS− was never paired with the US.

Physiological recordings during fear conditioning were undertaken with 5 mm inner diameter Ag/AgCl electrodes filled with non-hydrating electrodermal paste. Electrodes were fitted to the participants’ index and middle finger of the non-dominant hand. Signals were acquired at a sampling rate of 1000 Hz using a 16-bit BrainAmp ExG amplifier and Brain Vision Recorder software, version 1.2 (Brain Products GmBH, Gilching, Germany).

### Exposure treatment

Treatment comprised two sessions à 60 (±2) minutes of guided exposure, which was based on a modified version of the protocol by Öst^21^. A hierarchy of seven steps with increasing difficulty was used, ranging from watching the spider in a glass to having the spider walk over the arm [for details, see^22^]. Each step was first modeled by the experimenter, after which the participant performed the step himself and provided a fear rating (SUDS). The next step was initiated when fear had decreased to a SUDS of 30 or below. All steps of the hierarchy were initially accomplished with a vibrating spider (*Pholcidae*, 1 cm). Thereafter, all steps of the hierarchy were repeated with house spider (*Tegenaria domestica*, 1 cm). A session was terminated when either 60 minutes had elapsed or when all steps of the hierarchy with the house spider had been completed, whatever occurred first. Participants being able to complete all exposure steps within the predetermined time were classified as completers. Non-completers were classified as participants who were unable to accomplish all steps of exposure within the allotted time (based on^20^). A participant received completer status if he/she managed to accomplish the fear hierarchy with the house spider (note that a prerequisite for this hierarchy was mastering the hierarchy with the vibrating spider first), regardless of whether it took him/her one or two sessions. Only 3 participants, however, were able to accomplish both hierarchies at session 1. Those participants nevertheless returned to the lab for a post-assessment and were included in the analyses.

### Materials and measures

#### Control variables and diagnostic interview

Differences in depression, anxiety and stress were measured with selected items from the Depression Anxiety Stress Scales (DASS)^23^. Spider phobia (according to DSM-IV-TR criteria) was determined by a trained interviewer by means of the short diagnostic interview for mental disorders (Mini-DIPS)^24^.

#### Skin conductance responses (SCRs)

For SCR analyses (as part of fear conditioning), the maximum amplitude was calculated via a trough-to-peak analysis within a response window of 1–8 s after CS onset. All SCR data were logarithmized (using the natural logarithm) and range-corrected (SCR for each trial divided by the maximum logarithmized response to the US).

#### CS valence and CS-US contingency ratings

After each phase of the fear conditioning procedure, ratings of CS valence (“how do you feel when you hear this tone”) as well as CS-US contingency (“do you think that this tone is paired with an electrical stimulation?”) were obtained. These ratings were obtained on 5-point scales from −2 (valence: very uncomfortable; contingency: very unlikely) to +2 (valence: very comfortable; contingency: very likely), with 0 indicating neutrality (valence) or equiprobability (contingency).

#### Spider fear-related questionnaires

German versions of the Spider Phobia Questionnaire (SPQ)^25^, the Fear of Spiders Questionnaire (FSQ)^26^ and the Spider Phobia Beliefs Questionnaire (SBQ)^27^ were used, with higher scores indicating greater fear of spiders.

#### Behavioral approach test (BAT)

The BAT was used to measure fear and avoidance of a house spider (i.e., same as used for the exposure session). The spider was placed in a plastic container at the far end of the room. Participants were instructed to approach the spider as fast and close as possible until their fear becomes intolerable. BATs were scored from 0 (=refused to enter the room) to 10 (=touched the spider with the bare fingertip). Each participant was subjected to two BATs during the follow-up assessment (six weeks later). Here, the BAT was conducted in both the treatment context as well as in a novel context. The novel context was an unfamiliar room which differed in terms of furniture and interior decoration. Participants were subjected to the BAT in the therapeutic and novel context in a counterbalanced manner.

#### Subjective units of distress scale (SUDS)

During the exposure session and at the closest proximity to the spider during the BAT, the primary fear measure was the SUDS^28^, with scores ranging from 0 (=no fear) to 100 (=excessive fear).

### Statistical analyses

The *N* = 53 participants were divided into two groups (i.e., completers: *n* = 29; non-completers: *n* = 24) based on whether they had accomplished all steps of exposure within the allotted time. Data was analyzed in IBM SPSS, version 24 (Armonk, NY, USA). Since data at follow-up comprised only *N* = 46 participants (due to drop-out), pre- to post-treatment and post-treatment to follow-up data were analyzed separately. First, we analyzed whether exposure-induced improvement (pre- to post-treatment) and fear recovery (post-treatment to follow-up) differed in completers and non-completers using a series of mixed analyses of covariance (ANCOVA) on different measures of therapeutic success (i.e., BAT score, subjective fear during the BAT^1^, spider-fear related questionnaires). Time was entered as within-subjects factor and group (completers vs. non-completers) as between-subjects factor. The DASS depression score was used as a covariate (due to significant group differences at pre-treatment, see results). Contextualized fear at six week follow-up was analyzed with mixed ANCOVAs, using context (familiar vs. novel context) as within-subjects factor, group as between-subjects factor and the depression score as a covariate. Next, differences in fear conditioning data in completers and non-completers were analyzed with mixed ANCOVAs, conducted separately per conditioning phase (i.e., habituation, fear acquisition training, extinction training, retrieval test) and outcome measure. In each analysis, CS (CS+ vs. CS−) served as within-subjects factor, group (completers vs. non-completers) as between-subjects factor and the depression score as covariate. Regarding the retrieval test, context (retrieval context A vs. B) served as an additional within-subjects factor. SCR data were averaged across two trials per CS during habituation, five trials during fear acquisition training (yielding early vs. late acquisition), four trials during extinction training (yielding early vs. late extinction) and three trials during the retrieval test in each context. Hence, for fear acquisition and extinction training, block (early vs. late) was entered as another within-subjects factor. Significant main and interaction effects were followed by simple effects analyses. The effects of the covariate are only reported if significant. Results were considered significant at *p* < 0.05.

## Results

### Characteristics of completers and non-completers

Completers and non-completers were comparable in age (non-completers: *M* = 25.5, *SD* = 6.78; completers: *M* = 24.38, *SD* = 4.41; *t*_(38.09)_ = 0.7; *p* = 0.49), gender distribution (*n* female; non-completers: *n* = 22; completers: *n* = 27; *X*2_(df=1)_ = 0.04, *p* = 0.84), their total score on the DASS (non-completers: *M* = 15.85, *SD* = 13.77; completers: *M* = 10.24, *SD* = 7.28, *t*_(51)_ = 1.9, *p* = 0.063). Whereas there were no-significant differences between completers and non-completers on the anxiety (non-completers: *M* = 3.75, *SD* = 3.71; completers: *M* = 2.31, *SD* = 2.58, *t*_(51)_ = 1.66, *p* = 0.10) and stress (non-completers: *M* = 7.43, *SD* = 5.26; completers: *M* = 6.21, *SD* = 4.3, *t*_(51)_ = 0.93, *p* = 0.36) subscales of the DASS, groups differed on the depression subscale (non-completers: *M* = 4.67, *SD* = 5.9; completers: *M* = 1.72, *SD* = 1.91, *t*_(26.98)_ = 2.34, *p* = 0.027).

Importantly, completers finished all fourteen steps of the exposure hierarchy within *M* = 91.76 (*SD* = 24.09) minutes. By contrast, non-completers could only accomplish *M* = 8.17 (*SD* = 3.47) steps of the hierarchy within the allotted time (i.e., 120 min). In accord, completers had a better exposure efficiency score^29^, i.e., completed steps/exposure duration (non-completers: *M* = 0.07, *SD* = 0.03, completers: *M* = 0.17, *SD* = 0.06, *t*_(40.91)_ = 7.60, *p* < 0.001).

Groups did also not differ with regard to US valence prior to fear conditioning (non-completers: *M* = −1.8, SD = 0.38; completers: *M* = −1.7, *SD* = 0.47; *t*_(51.0)_ = 1.05, *p* = 0.3).

### Exposure success in completers and non-completers (pre-treatment to post-treatment)

**Figure 3:**
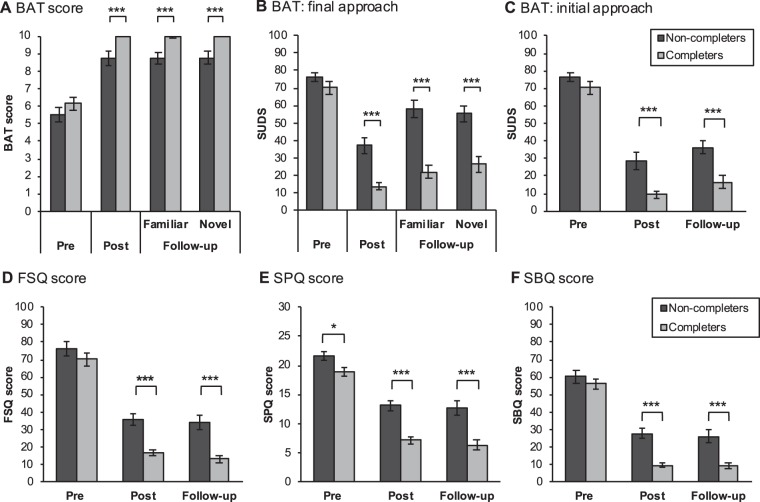
Exposure-induced changes in fear and avoidance (**A**–**F**) in completers and non-completers at pre-treatment, post-treatment and six-weeks follow-up. Results are displayed as means ± 1 SEM based on the raw data (i.e., not adjusted for the covariate). Asterisks indicate significant pair-wise comparisons with *p < 0.05., ***p < 0.001 at each assessment based on the model, in which the DASS score was entered as a covariate.

The results of the outcome measures of exposure success are displayed in Fig. 3A–C. Please note that *n* = 1 participant missed to provide a fear rating during the BAT at pre-treatment as well as *n* = 1 at the final and *n* = 4 at the initial approach distance of the BAT at post-treatment. Therefore, the mixed ANCOVAs on these outcomes are based on fewer degrees of freedom (i.e., due to pairwise deletion to handle missing data). As indicated by significant main effects for time, exposure was highly effective in promoting approach behaviour, decreasing subjective fear during the BAT at both approach distances and reducing scores on the spider-fear related questionnaires (all *F* > 132.18, *p* < 0.001). The main effect for group was significant across all outcome measures (all *F* > 6.56, *p* ≤ 0.013), in favor of completers (cf. Fig. 3). For subjective fear at the final approach distance as well as all spider fear-related questionnaires (but not for the BAT score and fear at the initial approach distance), these main effects were qualified by their interactions, indicating that the magnitude of exposure-based improvement from pre- to post-treatment (all *F* > 5.66, *p* ≤ 0.021) was greater in completers vs. non-completers.

Importantly, pairwise comparisons showed that completers had less avoidance, less fear during the BAT (both approach distances) and lower scores on the spider fear-related questionnaires at post-treatment (all *p* < 0.001), see Fig. 3.

#### Fear recovery (post-treatment to follow-up in familiar context)

We found no evidence for fear recovery on measures of the questionnaires and the BAT score from post-treatment to follow-up (main effects for time: all *F*_(1,43)_ < 2.79, *p* > 0.10). Whereas the same was true for self-reported fear at the initial approach distance (main effect for time: *F*_(1,40)_ = 2.86, *p* = 0.1), participants showed an increase in fear from post-treatment to follow-up at the final approach distance (main effect for time: *F*_(1,42)_ = 13.33, *p* = 0.001). The effects for time were not differentially modulated by group (all interactions with group: *p* > 0.06). As expected, in each analysis, the main effect for group was significant (all *F* > 19.02, *p* < 0.001), reflecting a better outcome at each assessment in completers vs. non-completers (all pairwise comparisons, *p* < 0.001; *cf*. Fig. 3).

#### Contextualized fear in the BAT at follow-up (familiar vs. novel context)

Participants underwent the BAT in both the therapeutic and novel context at follow-up. We found no evidence for contextualized fear on the behavioral (i.e., BAT score) and subjective level (i.e., fear at the final approach distance during the BAT; main effects for context: all *F*_(1,43)_ ≤ 0.21, *p* > 0.64) across participants. Changing the context did not affect groups differently (group x context interaction: all *F*_(1,43)_ ≤ 2.51, *p* > 0.1) across the BAT score and subjective fear. Only the main effect for group attained significance (all *F*_(1,43)_ > 19.18, *p* < 0.001), with less avoidance and subjective fear in both the therapeutic and novel contexts in completers vs. non-completers (all *p* < 0.001).

### Differential fear conditioning in completers and non-completers

**Figure 4:**
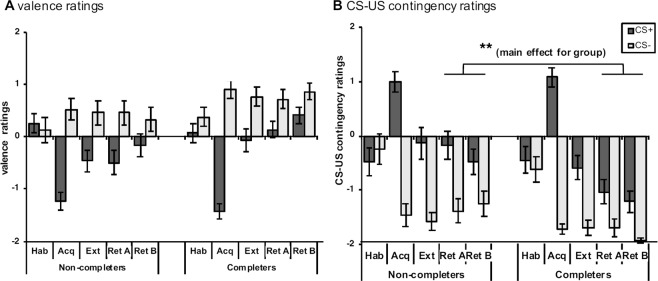
(**A**,**B**) Mean valence (**A**) and contingency (**B**) ratings after habituation (hab), acquisition (acq) in context A, extinction (ext) in context B, retrieval in context A (ret A) and retrieval in context B (ret B) are displayed separately for CS+ and CS−. Data is displayed as means ± 1 SEM based on the raw data (i.e., not adjusted for the covariate). Asterisks indicate the significant main effect for group with **p < 0.01 based on the mixed ANCOVA, in which the depression score was entered as the covariate.

**Figure 5:**
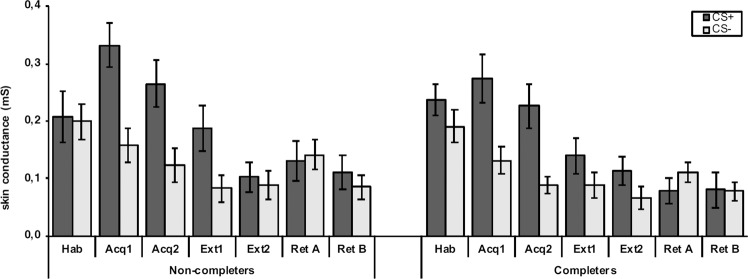
Mean skin conductance responses are shown for the phases habituation (hab), first and second block of acquisition (acq 1 and acq2, respectively) in context A, first and second block of extinction (ext1 and ext2, respectively) in context B, retrieval in context A (ret A) and retrieval in context B (ret B) separately for CS+ and CS−. Data is represented as raw data and indicate means ± 1 SEM.

The results of fear conditioning within completers and non-completers are displayed in Figs. 4A,B and 5.

#### Habituation

There were no significant main or interaction effects across measures of CS valence, CS-US contingency and SCRs (all *F*_(1,50)_ < 2.13, *p* > 0.15).

#### Fear acquisition

After fear acquisition training, participants rated the CS+ as more unpleasant (main effect CS: *F*_(1,50)_ = 62.72, *p* < 0.001) and as more likely to be followed by the US (main effect CS: *F*_(1,50)_ = 101.28, *p* < 0.001) compared to the CS−, indicating successful fear acquisition. Likewise, higher SCRs were found towards the CS+ relative to the CS− (main effect CS, *F*_(1,50)_ = 31.93, *p* < 0.001). In addition, SCRs habituated from the first to the second block (main effect block, *F*_(1,50)_ = 16.81, *p* = 0.001). This main effect for block was further modulated by the depression score (interaction: *F*_(1,50)_ = 4.33, *p* = 0.043). Main effects for group and any interaction effects were non-significant.

#### Extinction

The main effect for CS remained significant across measures of CS valence and CS-US contingency, but nor for SCRs: Relative to the CS−, participants rated the CS+ as more unpleasant (*F*_(1,50)_ = 9.67, *p* = 0.003), attributed higher CS-US contingency ratings towards the CS+ (*F*_(1,50)_ = 30.51, *p* < 0.001). However, they just ceased to show elevated SCRs (*F*_(1,50)_ = 0.053) in response to the CS+. No other main or interaction effects were significant.

#### Retrieval

The main effect for CS remained significant for CS valence (*F*_(1,50)_ = 8.64, *p* = 0.005) and CS-US contingency (*F*_(1,50)_ = 28.69, *p* < 0.001), but not for SCRs (*F*_(1,50)_ = 1.24, *p* = 0.27). There was, however, an interaction between CS-type and the depression score (*F*_(1,50)_ = 10.85, *p* = 0.002). Interestingly, as indicated by significant main effects for group, non-completers attached a more negative valence to the CSs (*F*_(1,50)_ = 4.45, *p* = 0.04) and higher CS-US contingency ratings (*F*_(1,50)_ = 9.52, *p* = 0.003) relative to completers.

To examine whether the main effects for group on measures of CS valence and CS-US contingency were due to pre-existing group differences at the end of extinction, additional 2 × 2 × 2 mixed ANCOVAs with the depression score as the covariate, phase (extinction, retrieval) and CS-type as within-subjects factors and group as between-subjects factor were conducted separately context A and context B. As expected, the main effect for CS was significant in all analyses (all *p* ≤ 0.006). Interestingly, we found that the decline in CS-US contingency ratings from extinction to retrieval in context B was modulated by group, with a more pronounced decline in completers (phase x group interaction: *F*_(1,50)_ = 4.81, *p* = 0.033; group: *F*_(1,50)_ = 5.89, *p* = 0.019). Precisely, whereas completers and non-completers were comparable in their average contingency ratings by the end of extinction, completers had significantly lower contingency ratings after retrieval. By contrast, the main effect for group (*F*_(1,50)_ = 4.24, *p* = 0.045) was not qualified by a phase x group interaction (*F*_(1,50)_ = 2.03, *p* = 0.16) in the analysis involving context A. None of the aforementioned effects were attained for CS-valence ratings and no other main or interaction effects were observed in all analyses.

## Discussion

In the present study, we showed that fear extinction (retrieval) is related to the ability to complete exposure in a predetermined time and exposure therapy outcome. Participants who mastered the exposure in a predetermined time (completers) displayed an enhanced short- and long-term exposure treatment outcome. The latter effect was found across all subjective fear measures at post-treatment and at follow-up. Completers also showed more increase in approach behavior during the BAT relative to non-completers. However, we did not find this time x group interaction for the BAT scores. This suggests that pre-existing group differences might confound the time x group interaction effects at the behavioral level. The absence of a significant time x group interaction is probably due to ceiling effects, since the majority of participants succeeded to attain the highest BAT score after the exposure, irrespective of group assignment. Although it was recently shown that a more challenging exposure can lead to a better treatment outcome (at least at the level of fear ratings^20^), the findings of the present study suggest that exposure therapy outcome also depends on the ability to complete all exposure steps in a fixed time. One important implication of this finding is that in conditions, in which patients are unable to finish all exposure steps in a predetermined time, the short- and long-term beneficial effects are reduced. However, our data also indicate that such a reduced symptom improvement is not reflected on all therapy outcome measures equally.

Most importantly, we showed that specific changes in fear conditioning are related to the ability to finish the exposure in a predetermined time and consequently to less-pronounced symptom improvement. Non-completers showed no changes in fear acquisition and the early phase of extinction training relative to completers. However, higher CS-US contingency ratings during extinction (retrieval) was evidenced in non-completers as compared to completers. The latter finding can be interpreted as a deficient processing of CS-US contingency during extinction (retrieval) in non-completers. Notably, this effect was restricted to CS-US contingency ratings. Contrary to our hypothesis, we did not observe a context-dependent renewal of fear responses in the extinction retrieval phase. Our context-dependent fear conditioning task was adapted from Alvarez *et al*.^30^ who used auditive CSs and visual VR-based contexts. While the findings in the Alvarez *et al*.^30^ clearly indicate a contextual renewal effect on the level of SCR and CS-US contingency ratings, we could not replicate this effect. The absence of a significant fear renewal is surprising. One reason might be that the use of a phobic sample (instead of healthy participants, see^30^) yielded different performance signatures in the task due to changes in the processing of CS and/or contextual information in phobic relative to healthy participants. Alternatively, slight differences in the procedures, such as the introduction of a habituation phase, might account for the absence of significant fear renewal effects. The entire experimental procedure was conducted in one session with relatively short delays between the habituation, acquisition, extinction, and extinction retrieval phases. The assessment of fear acquisition, extinction and extinction retrieval in one session (versus an experimental procedure which employs two or three days) and the use of relative short delays might have led to a sensory habituation process to the CS stimuli^31^ and the absence of fear renewal. Furthermore, CS valence and contingency ratings were not assessed trial-by-trial but were taken after the respective phase. Consequently, the participants were able to retrieve that the preceding CS+/CS− presentation in the retrieval phase did not differ between the contexts. This retrospective assessment of CS-US valence and contingency ratings could explain why CS-US valence and contingency ratings did not differ in the contexts A and B during the retrieval phase.

The short delay between the extinction and retrieval phase might have influenced our core findings. The interpretation that the groups differed in extinction retrieval is unlikely due to several reasons. First, as already stated, the retrieval phase was conducted shortly after the extinction phase. Secondly, the retrieval phase was not further modulated by context. It is thus rather possible that the participants continued to process the recently established CS-US associations. In accordance with this interpretation, we observed a further decline of CS-US contingency ratings from the late extinction phase to the extinction retrieval phase in both contexts. In other words, participants from both groups continued to process the new CS–US contingencies from the extinction phase in the retrieval phase. Notably, the further decline in CS-US contingency ratings from the extinction to the retrieval phase in context B (i.e., the extinction context) was stronger in completers. The latter suggests that non-completers were not able to process the new CS–US contingencies as efficiently as completers. Such a pattern of findings could potentially explain why non-completers show difficulties to complete all exposure steps, e.g. due to the failure to further extinguish US expectancy to the CSs. Future studies should employ longer delays between extinction training and retrieval (e.g. >24-hours) to examine whether completers and non-completers differ with respect to extinction retention performance.

Nevertheless, one important implication of the present finding is that the exposure session duration and or frequency need to be increased in non-completers. Extending the exposure duration or frequency in non-completers could be effective to further reduce aversive expectancies of the phobic stimulus. Alternatively, the selective promotion of contingency learning during exposure might help to expedite treatment duration and efficacy in non-completers. Interestingly, contingency instructions can alter the course of extinction for individuals with high intolerance of uncertainty^32^. It remains to be determined how contingency learning can be promoted in the exposure context.

Exposure therapy implementation and success in routine care is highly dependent on the organizational and economic demands. In general, the duration and frequency of exposure sessions is fixed, which can bear some difficulties to design appropriate treatment schedules in clinical care settings (see^6^). Shortening treatment frequency and duration by designing individualized treatment regimens can decrease treatment costs and further promote the transfer of exposure treatment to routine care^7^. However, the present results implicate that some anxiety patients might need longer treatment durations and/or higher frequencies to complete all exposure steps. In the absence of additional time or appropriate add-on interventions, short- and long-term effects of therapy are less pronounced.

Our study illustrates how research on variability in exposure therapy duration and outcome may profit from systematic research on fear extinction and retrieval^33^. Previous studies showed a mutual relationship between differences in extinction learning and variability in cognitive therapy outcome in children^12^, variability in outcomes of an exposure therapy analog^13^ and variability in anxiety reductions after exposure in patients with social anxiety^14^. The present study extends these findings to treatment-seeking adults with specific phobia [but see^12,14^]. The use of a standardized exposure session [but see^13^] in the absence of other additional cognitive-therapeutic interventions [but see^12^] allows to exclude the possibility that other therapeutic elements [e.g., cognitive reappraisal, see^34^] confounded the functional link between extinction and exposure treatment-related measures. Our findings are partially comparable to the findings by Forcadell *et al*.^13^. Similar to our approach, Forcadell *et al*. assessed fear learning and extinction on different levels and showed that these measures differ with respect to the predictive value. Interestingly, US expectancies had a better predictive value than SCR. This is comparable to our finding of an impairment in CS-US contingency processing during extinction (retrieval) in non-completers. However, it must be noted that in general great heterogeneity in data extraction, calculation and interpretation of conditioning data exists. This might reflect existing conceptual and procedural challenges in human fear conditioning research. For instance, at least 16 different operationalizations of the extinction retention index exist while correlation coefficients among these different operationalizations as well as among measures of fear/anxiety show a wide range of variability^15^. There is certainly a need for more standardization in fear conditioning research^33^.

In the present study, we attempted to examine the link between fear conditioning and fear renewal after successful exposure. Since fear renewal after extinction might be analogous to a context-dependent increase in fear responding after exposure^35–37^, we hypothesized a more pronounced contextualized fear responding at follow-up in non-completers. Yet, although non-completers showed less therapy-induced changes on the level of self-report measures at follow-up in both the familiar and novel contexts, this effect was not attributable to an increased context-dependent recovery of fear. Precisely, changing the contextual details during the BAT at follow-up was not associated with a more pronounced fear renewal in both groups. These findings contrast previous studies using similar therapy protocols, which found significant context-dependent increases in fear responding using similar follow-up durations^35–38^. One potential explanation for the absence of fear renewal might be the operationalization of the contextual difference between the two BATs. In fact, the demonstration of a significant fear renewal critically depends on the amount of contextual change and the fear indices (subjective or psychophysiological) used to detect context-dependent increases in fear^35–37^. Future studies might incorporate longer follow-up periods to derive possible associations between extinction retrieval and fear recovery after exposure [e.g.,^38^]. Furthermore, changing the experimenter in addition to the visual contextual details during the BAT at follow-up as well as the simultaneous measurement of different fear indices might aid in detecting significant fear renewal in the exposure setting.

Collectively, our findings are partially in line with observations from similar studies assessing the prognostic validity of fear extinction on exposure therapy outcome^13^. We showed that the failure to further extinguish US expectancy to the CSs during fear extinction (retrieval) is linked to an inability to accomplish exposure in a fixed time and to less treatment-induced gains. The characterization of extinction learning and retrieval performance thus may offer sound therapeutic advice whether a particular treatment regimen will be effective when given in a standardized form to a particular patient. Second, it justifies the application of specific mechanism-based strategies to expedite exposure-induced symptom improvement based on the fear extinction model^39,40^. Patients who do not profit from a standardized therapy setting (e.g., because they are unable to complete all exposure steps in a predetermined time) might attain significant treatment gains when being treated with additional interventions under the same treatment regimen. Ultimately, the identification of interventions which are suitable to increase extinction (which in turn could potentially decrease treatment duration and costs) would be highly valuable to improve standardized exposure in routine practice. Evidence from preclinical studies support the hypothesis that individual differences in rate-of-extinction are strongly related to vulnerability to relapse^41^.

Study limitations include the relatively homogenous sample of spider phobic individuals, thus further precluding the generalization of findings to other anxiety disorders. Moreover, the absence of fear renewal after exposure warrants further investigation on the link between fear extinction retrieval and generalization of treatment effects. Future studies should employ longer delays between fear acquisition and extinction training and/or extinction training and retrieval, respectively. Finally, in the present study we separated participants into completers and non-completers (similar to the approach by^12^ who classified participants into responders and non-responders) and looked at possible differences in the pattern of fear conditioning/extinction performance. This was done due to considerations from local routine clinical care settings (where exposure session typically last 60 minutes) in addition to the need to reduce treatment-associated costs. Interestingly, other potential conditions exist which might hinder the implementation of longer exposure sessions (>60 min) in routine clinical care settings (see^6^). An alternative strategy could be to use the actual time spent before completing the exposure task as a continuous variable in the respective analyses. Future studies investigating the prognostic value of fear conditioning measures on exposure therapy processes and outcome should be pre-registered. The same applies to our study which might limit our general approach and conclusions.

Nevertheless, our data suggest a potential link between extinction learning and efficacy of exposure treatment in anxiety disorders. Our findings might aid in developing personalized treatment regimens which can decrease treatment-associated costs and expedite exposure-induced symptom improvement.

## Data Availability

The datasets generated during and/or analyzed during the current study are available from the corresponding author on reasonable request.
